# Hypophosphataemic Rickets Secondary to Raine Syndrome: A Review of the Literature and Case Reports of Three Paediatric Patients' Dental Management

**DOI:** 10.1155/2021/6637180

**Published:** 2021-01-07

**Authors:** Lorna Hirst, Gehan Abou-Ameira, Simon Critchlow

**Affiliations:** Dental and Maxillofacial Department, Great Ormond Street Hospital, Great Ormond Street, WC1N 3JH, UK

## Abstract

Raine Syndrome (RS) also referred to as lethal osteosclerotic bone dysplasia describes an exceptionally rare autosomal recessive disorder with an estimated prevalence of <1 in 1,000,000. Endocrinological manifestations such as hypophosphataemic rickets depict a recent finding within the phenotypic spectrum of nonlethal RS. The dental sequelae of hypophosphataemic rickets are significant. Spontaneous recurrent abscesses on noncarious teeth result in significant odontogenic pain and multiple dental interventions. The dental presentations of nonlethal RS are less widely described within the literature. Amelogenesis Imperfecta (AI), however, was recently postulated as a key characteristic. This article presents the dental manifestations and extensive restorative and oral surgical intervention of three siblings with hypophosphataemic rickets secondary to Raine Syndrome treated at Great Ormond Street Hospital for Children, a tertiary referral hospital.

## 1. Introduction

Raine syndrome (RS) (OMIM # 259775) is an especially rare autosomal recessive disorder with an estimated prevalence of <1 in 1,000,000 [1, 2]. Endocrinological manifestations such as hypophosphataemic rickets are a newly described feature within the phenotypical plethora of RS [3]. Dental sequelae of hypophosphataemic rickets are significant, broadly encompassing multiple spontaneous dental abscesses in noncarious teeth, dentine and enamel defects, and dental anomalies [4–7]. The dental presentation in RS is less frequently described in the literature.

RS is commonly referred to as lethal osteosclerotic bone dysplasia due to high mortality in the neonatal period [8]. More recently, a milder phenotype has been described that is compatible with life [1–3]. RS characteristically presents with exophthalmos, cerebral calcifications, choanal atresia, and osteosclerosis [2, 8]. Exorbitism and midfacial hypoplasia often present as a Crouzon-like facial appearance. Heterogeneity is considerable amongst the nonlethal type, and a paucity of data results in the possibility of the complete phenotypic spectrum not being delineated [1–3]. Genetically, mutations in the FAM20C gene on chromosome 7, which encodes a Golgi Casein Kinase that phosphorylates fibroblast growth factor 23 (FGF23), account for RS, with a high frequency of consanguineous parents in affected patients [2, 3, 9, 10].

Hypophosphataemic rickets is a disorder of the growing skeleton, characterised by hypophosphatemia, impaired intestinal absorption of calcium, and rickets that is resistant to vitamin D [11, 12]. Phosphate is the main component of hydroxyapatite (Ca_10_(PO_4_)_6_(OH)_2_) and essential in cell metabolism (adenosine triphosphate (ATP)) [11]. Homeostasis of serum phosphate levels are controlled by renal absorption, bone resorption, and hormonal (calcitriol, parathyroid hormone (PTH), and fibroblast growth factor 23 (FGF23)) regulation of intestinal absorption [5, 11]. Subsequent hypophosphatemia, thus, impairs mineralisation of the teeth, bones, and cartilage [5, 13].

Clinical features include bowing of lower extremities, disproportionately short stature, cranial osseous deformities, and dental manifestations [5, 12, 14]. The dental presentation is significant and associated with a lifelong burden of restorative and oral surgical intervention. The most common clinical presentation is multiple recurrent dental abscesses in noncarious teeth, and in some patients, this may be the first clinical manifestation of their disease [4, 5]. Defects in dentine mineralisation, thin, hypoplastic enamel, enlarged pulp chambers extending to the enamel–dentine junction (EDJ), and root resorption are commonly cited [4–6, 11, 14, 15].

## 2. Case Descriptions

This paper describes three siblings, 14- and 13-year-old males and an 11-year-old female, of Somalian descent. Their parents are second cousins. The siblings had a diagnosis of hypophosphataemic rickets secondary to Raine Syndrome caused by homozygous mutation in FAM20C gene [FAM20C. C (1094G > T); C (1094G > T). P. (Gly365Val); and (P. (Gly365Val)]. A summary of their medical and dental management can be seen in Table 1.

### 2.1. Case One

Case one describes a 14-year-old male who initially presented to Great Ormond Street Hospital with nystagmus and raised intracranial pressure associated with a sagittal synostosis. This was treated with a posterior vault expansion at the age of 2. On clinical examination, he had a convexity over the closed anterior fontanelle, a high anterior hairline, mild exorbitism, midfacial hypoplasia, narrow nose, and high narrow palate. He also had generalised osteosclerosis. His body proportions and limbs were only mildly affected.

Dentally, he was first treated at the age of seven, when he presented with spontaneous abscesses of the maxillary central incisors, left lateral incisor, and left first permanent molars requiring extraction under general anaesthetic (GA). At the age of eight, further abscesses of the mandibular incisors and maxillary left first premolar occurred, which were also extracted under GA. He later presented with additional abscesses on the maxillary right first premolar at the age of nine, which was extracted under local anaesthetic (LA). Later that year, abscesses also presented on all mandibular premolars. These teeth were surgically extracted under GA along with the mandibular left canine. At the age of ten, further abscesses presented and the decision to complete a full clearance under GA was made. Prosthetic intervention included provision of sequential complete dentures (C/C) to account for the growth. In the future, dental implants may be considered to enhance the stability and retention of the prostheses.

### 2.2. Case Two

Case two depicts a 13-year-old male. He presented with sagittal and lambdoid craniosynostosis with raised intracranial pressure at the age of 15 months, which was treated with biparietal vault expansion. Bilateral intracranial calcification was also diagnosed of the globi pallidi, right corona radiata, and left parietal lobes. On clinical examination, he had a turricephalic head shape with a tall, bossed forehead, high anterior hairline, and convexity over the closed anterior fontanelle. Hypotelorism, down-slanting palpebral fissures and midfacial hypoplasia with a high narrow palate, midline raphe, and wide uvula were also present. Additionally, he had generalised osteosclerosis, a barrel-shaped chest, spinal hyperlordosis, and scoliosis, and leg bowing was apparent with joint laxity. Furthermore, he had obstructive sleep apnoea (OPA), which was treated with Bilevel Positive Airway Pressure (BiPAP); in addition, he received an adenoidectomy at the age of 6.

Dentally, at the age of three, he presented with multiple spontaneous abscesses in the primary dentition. He required a GA for extraction of all primary first and second molars and primary maxillary canines. The first permanent molars were spontaneously abscessed at the age of six and were also extracted under GA. At the age of nine, he presented with significant odontogenic pain and further abscesses. Radiographically, bilateral cysts were noted in the mandible. Treatment options for multiple root canal treatments (RCTs) or extractions were discussed with the family, who opted for a full clearance at this time. This was completed under GA along with enucleation of the mandibular cysts. Removable prosthesis was subsequently constructed.

### 2.3. Case Three

Case three presents the youngest sibling, an 11-year-old female. Craniofacially, she presented with sagittal craniosynostosis and metopic ridging. Additionally, bilateral intracranial calcification was evident of the globus pallidi and scattered throughout both cerebral hemispheres. There were no concerns about raised intracranial pressure. Clinically, she also had a convexity over the closed anterior fontanelle, a high anterior hairline, mild exorbitism, midfacial hypoplasia, narrow nose, and high narrow palate. She also had generalised osteosclerosis, short stature, and leg bowing. Additionally, she had hypertension with mild renal impairment.

Dentally, she initially presented with multiple recurrent abscesses requiring repeat courses of amoxicillin. She received a general anaesthetic (GA) at the age of seven, for extraction of all primary first and second molars and primary maxillary canines. At the age of eight, she presented with a spontaneous abscess of mandibular right central incisor and had first-stage root canal therapy under local anaesthetic. At the age of nine, she presented with a retained root of the maxillary right primary second molar, which was extracted under local anaesthetic (LA). At the age of ten, she presented with draining sinuses and pulp exposure of the maxillary left central incisor. Treatment options were attempt root canal treatment (RCT) of mandibular central incisors, left lateral incisor, and maxillary left central incisor and extractions of maxillary left second premolar and first permanent molar or full clearance and provision of a prosthesis.

## 3. Discussion

Metabolic bone disorders, such as hypophosphataemic rickets, pose a relatively new characteristic of Raine Syndrome (RS) [1, 3]. Biallelic variations in the FAM20C gene provide the genetical basis of RS [1, 2]. FAM20C is essential for biomineralization and encodes the human homolog of dentine matrix protein 4 (DMP4), which is highly expressed in odontoblasts and somewhat in bone, where it is postulated to have a role in mineralisation [1, 3]. Mutations in dentine matrix protein 1 (DMP1) have been established in the aetiology of inherited forms of hypophosphataemic rickets (HHR) [1]. Further research is warranted, although it is anticipated that the roles of DMP4 and DMP1 are comparable, thus hypothesising a link between Raine Syndrome and hypophosphataemic rickets [1, 3]. A comparison of the clinical, radiographic, and histological features of RS and HHR are depicted in Table 2.

The dental presentations of the three siblings are complex and significant, overlapping the phenotypic spectra of both HHR and RS described within the literature. Notably, features specific to RS were evident, such as high-vaulted palate and radiographic lack of distinction between enamel and dentine. Characteristics implying HHR were also present, such as recurrent spontaneous dental abscesses, sinus tracts, spontaneous loss of vitality, enlarged pulp horns, and taurodontism. Overarching and interrelated features across both RS and HHR included generalised hypoplastic, yellow-brown enamel, malocclusion, and root hypoplasia. The paucity of data regarding the complete dental phenotypical spectra of nonlethal RS, combined with the superimposed infrequent finding of hypophosphataemic rickets, makes it challenging to ascertain with a great degree of confidence the precise aetiology of the dental anomalies within the three siblings.

A presenting complaint of multiple recurrent abscesses associated with noncarious teeth is frequent in certain patients with hereditary hypophosphataemic rickets and was the underlying presentation in our cases. Provision of root canal treatment (RCT) in such patients can be complicated by atypical pulpal morphology and root hypoplasia [4, 6, 14]. The propensity towards endodontic failure and recurrent abscess formation may be attributed to reinfection of the root canal system due to dentinal defects [4]. Preventative measures such as oral hygiene instruction, dietary advice, and fluoride varnish application (22,600 ppm) are indispensable in reducing the burden of dental disease.

Whether medical treatment of hereditary hypophosphataemic rickets can prevent or expressively reduce the characteristic dental phenotype is a contentious issue in the medical literature. Prevention of dentinal effects in the primary dentition is evidently largely unfeasible since dentinogenesis of the primary dentition occurs in utero. The provision of medication postnatally, however, theoretically has the potential to beneficially affect the formation of permanent dentinal hard tissues. Conventional treatment regimens for HHR may include oral phosphate supplementation and activated vitamin D, such as calcitriol. Since inorganic phosphate and calcium are fundamental for dental mineralisation in a similar way to bone mineralisation, a link between hypophosphatemia and aberrations in dental structure is clearly discernible [6]. Several studies have demonstrated phosphate supplements reducing irregularities in mineralisation or even curing dental anomalies [6]. Contrarily, other studies debate the role of medical therapy in the correction of dental calcification [4, 6, 16], whilst others suggest that conventional therapy may be preferable to newer therapies, such as Burosumab, in reducing dental effects in HRR [4, 6, 16].

The paucity of cases of Raine Syndrome, particularly the nonlethal phenotype and challenges in management, precludes the ability to definitively determine the relationship between disease progression and dental manifestations. To further complicate matters, the complete dental phenotype of Raine Syndrome is unlikely fully delineated. Additionally, certain dental manifestations, notably, amelogenesis imperfecta and cleft lip and palate, have the potential to be superimposed dental features in some patients, rather than related to the underlying Raine Syndrome diagnosis.

Raine Syndrome is an extremely rare inherited disorder with amelogenesis imperfecta and ectopic tissue calcifications being common dental manifestations. The dental phenotype of hereditary hypophosphataemic rickets is, however, more widely described within the literature. The precise aetiology of the dental anomalies in our patients may be attributable to hypophosphatemia during dentinogenesis or primarily due to mutations in the FAM20C gene itself. The paucity of cases of nonlethal Raine Syndrome means that dental phenotypical spectrum may not be fully known. Independent of the exact aetiology of the enamel–dentine defects present in these three patients, the dental management follows the overarching principles of comprehensive prevention, early control of infection, pulpal protection, and long-term restorative stability.

## Figures and Tables

**Table 1:** A summary of the genetic, medical, and dental diagnoses of the three siblings and their dental management.

	Genetic diagnosis	Clinical phenotype	Medical management	Dental diagnoses and management
Fourteen-year-old male (case 1)	Hypophosphataemic rickets secondary to Raine Syndrome homozygous mutation in FAM20C	Sagittal synostosis	*Medications*	*Dental diagnosis*
		Raised intracranial pressure	Alfacalcidol 400 ng daily	Multiple periapical abscess with sinus tract
		Convexity; anterior fontanelle	Phosphate 2x daily	
		High anterior hairline		*Dental management*
		Midface hypoplasia	*Surgical management*	C/C provision (age 14)
		Exorbitism	Posterior vault expansion	Planned XLA: maxillary left canine, mandibular right central incisor
		Nystagmus		C/C provision (age 12)
		Nasal obstruction	*Medical management*	Full clearance (age 10)
		Narrow nose	BiPAP	XGA mandibular premolars (surgical), mandibular left canine (conventional) (age 9)
		High narrow palate		XLA maxillary right first premolar (age 9)
		Generalised osteosclerosis		XGA maxillary left first premolar, mandibular incisors (age 8)
		Obstructive sleep apnoea		XGA maxillary central incisors and left lateral incisor, left first permanent molars, incision, and drainage of abscess (age 7)

Thirteen-year-old male (case 2)	Hypophosphataemic rickets secondary to Raine Syndrome homozygous mutation in FAM20C	Sagittal and lambdoid craniosynostosis	*Medications*	*Dental diagnoses*
		Raised intracranial pressure	Alfacalcidol 400 ng daily	Multiple Periapical Abscess with Sinus Tract
		Bilateral intracranial calcification	Phosphate 2x daily	Bilateral Mandibular Cysts
		Turricephalic head shape	*Surgical management*	*Dental management*
		Bossed forehead	Biparietal vault expansion	C/C provision (age 13)
		High anterior hairline	Adenoidectomy	XLA maxillary left central incisor retained root^*∗*^ (age 12)
		Convexity; anterior fontanelle; midfacial hypoplasia	Spinal fusion	Enucleation of mandibular cysts (age 9)
		Hypotelorism	*Medical management*	XGA full dental clearance (age 9)
		Down-slanting palpebral fissures	Bilevel Positive Airway Pressure (BiPAP)	XGA first permanent molars (age 6)
		High narrow palate, midline raphe, and wide uvula		XGA maxillary primary canines and molars, mandibular primary molars (age 3)
		Generalised osteosclerosis		
		Barrel-shaped chest		
		Spinal hyperlordosis		
		Scoliosis		
		Leg bowing		
		Joint laxity		
		Atrophic rhinitis		
		Hypertrophy of adenoids		
		Choanal stenosis		
		Learning difficulties		

Eleven-year-old female (case 3)	Hypophosphataemic rickets secondary to Raine Syndrome (homozygous mutation in [FAM20C. C (1094G > T); C (1094G > T). P. (Gly365Val); and (P. (Gly365Val)]	Sagittal craniosynostosis	*Medications*	*Dental diagnoses*
		Metopic ridging	Alfacalcidol 400 ng daily	Dental caries
		Bilateral intracranial calcification	Amlodipine 10 mg	Chronic gingivitis
		Convexity; anterior fontanelle	Sodium acid, phosphate 1.936 g	Multiple periapical abscesses with sinus tract
		High anterior hairline	Vitamin D	Perio-endo lesion
		Midfacial hypoplasia		Dens in dente
		Exorbitism	*Surgical management*	Pulp exposure
		Narrow nose	Adenotonsillectomy	
		High narrow palate		*Dental management*
		Benign hypermobility syndrome	*Medical management*	First-stage RCT (GA) mandibular left incisors, right central incisor, and maxillary left central incisor, XGA maxillary left second premolar and first permanent molar (age 10)
		Generalised osteosclerosis	Echocardiogram, antihypertensives	XLA maxillary right second primary molar retained roots (age 9)
		Short stature	Dimercaptosuccinic acid (DMSA) scan	First-stage root canal treatment (LA) mandibular right central incisor (age 8)
		Leg bowing	Salbutamol and Beclomethasone inhalers	XGA maxillary primary canines and molars, mandibular primary molars (age 4)
		Asthmatic bronchitis	Intermittent antibiotics	Amoxicillin (multiple courses)
		Atopic rhinitis	Regular orthopaedic reviews	
		Hypertrophy of adenoids		
		Agranulocytosis		
		Hypertension with mild renal impairment		
		Learning difficulties		

^*∗*^Retained root, LA: local anaesthetic, GA: general anaesthetic, RCT: root canal treatment, C/C: complete denture, XGA: extraction under general anaesthetic, and XLA: extraction under local anaesthetic.

**Table 2:** The clinical, radiographic, and histological features of Hereditary Hypophosphataemic Rickets (HHR) and Raine Syndrome (RS) as described in the literature [2, 4–9, 11, 14, 15].

Diagnosis	Clinical features	Radiographic features	Histological features
Hereditary Hypophosphataemic Rickets (HHR)	Recurrent spontaneous dental abscesses, spontaneous loss of vitality, sinus tracts, eruption anomalies, defective dentine mineralisation, yellow-to-brown enamel hypoplasia, increased periodontal disease, malocclusion	Root resorption, pulp horns extending to enamel–dentine junction (EDJ), taurodontism, poorly defined lamina dura, hypoplastic alveolar ridge	Tubular dentinal clefts, increased and hypomineralised interglobular dentine, reduced secondary dentine, widened predentine, microclefts in enamel surface

Raine Syndrome (RS)	Hypoplastic amelogenesis imperfecta (AI), delayed dental eruption, high-vaulted palate, cleft palate, malocclusion, gingival enlargement, thin, yellow, and translucent enamel, incisal notch of central incisors	Ectopic eruption, pulpal calcifications, root hypoplasia, periapical pathology, lack of differentiation between enamel and dentine	Gingival or follicular calcifications, increased interglobular dentine, thin enamel

## Data Availability

Data sharing was not applicable to this article as no datasets were generated or analysed in the production of the manuscript.

## Supplementary Materials

Supplementary Materials
*Case 1*. Complete prosthesis at the age of 14. *Case 2*. Dentition at the age of 5. *Case 2*. Extra-oral midface hypoplasia at the age of 5. *Case 3*. Abnormal pulpal pathology, root hypoplasia, and poor discrimination between enamel and dentine radiographically. *Case 3*. Dentition at the age of 11.
