# Supplementary figures and images for: Hypophosphataemic Rickets Secondary to Raine Syndrome: A Review of the Literature and Case Reports of Three Paediatric Patients' Dental Management
Source: Case Rep Pediatr. 2021 Jan 7;2021:6637180. doi: 10.1155/2021/6637180 (PMC7808805; doi:10.1155/2021/6637180)

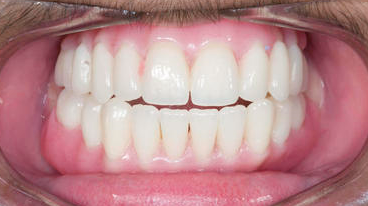

Supplement: Supplementary Materials — Case 1. Complete prosthesis at the age of 14. Case 2. Dentition at the age of 5. Case 2. Extra-oral midface hypoplasia at the age of 5. Case 3. Abnormal pulpal pathology, root hypoplasia, and poor discrimination between enamel and dentine radiographically. Case 3. Dentition at the age of 11. [file 6637180.f1.zip › 6637180.f1/Case 1 Complete Prosthesis aged 14.png]

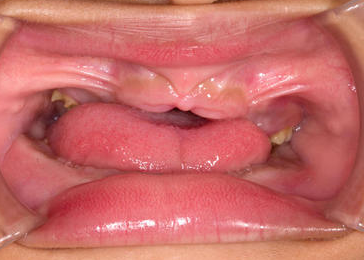

Supplement: Supplementary Materials — Case 1. Complete prosthesis at the age of 14. Case 2. Dentition at the age of 5. Case 2. Extra-oral midface hypoplasia at the age of 5. Case 3. Abnormal pulpal pathology, root hypoplasia, and poor discrimination between enamel and dentine radiographically. Case 3. Dentition at the age of 11. [file 6637180.f1.zip › 6637180.f1/Case 2 Dentition aged 5.png]

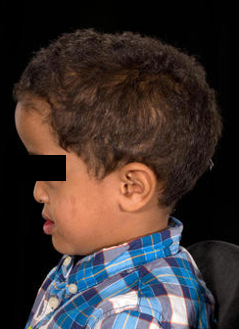

Supplement: Supplementary Materials — Case 1. Complete prosthesis at the age of 14. Case 2. Dentition at the age of 5. Case 2. Extra-oral midface hypoplasia at the age of 5. Case 3. Abnormal pulpal pathology, root hypoplasia, and poor discrimination between enamel and dentine radiographically. Case 3. Dentition at the age of 11. [file 6637180.f1.zip › 6637180.f1/Case 2 Extra-Oral Midface Hypoplasia aged 5.png]

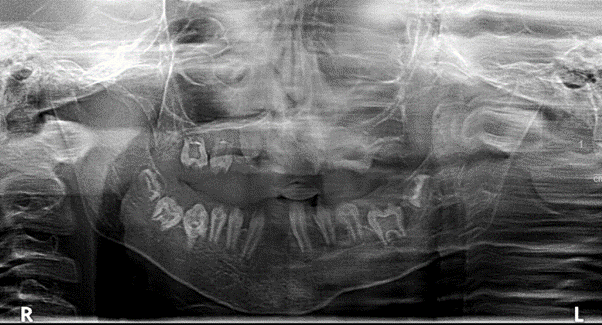

Supplement: Supplementary Materials — Case 1. Complete prosthesis at the age of 14. Case 2. Dentition at the age of 5. Case 2. Extra-oral midface hypoplasia at the age of 5. Case 3. Abnormal pulpal pathology, root hypoplasia, and poor discrimination between enamel and dentine radiographically. Case 3. Dentition at the age of 11. [file 6637180.f1.zip › 6637180.f1/Case 3 Abnormal pulpal pathology, root hypoplasia and poor discrimination between enamel and dentine radiographically..png]

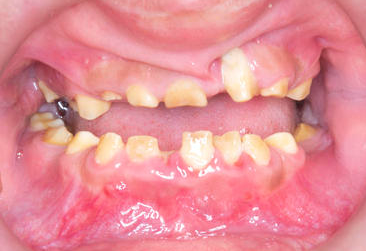

Supplement: Supplementary Materials — Case 1. Complete prosthesis at the age of 14. Case 2. Dentition at the age of 5. Case 2. Extra-oral midface hypoplasia at the age of 5. Case 3. Abnormal pulpal pathology, root hypoplasia, and poor discrimination between enamel and dentine radiographically. Case 3. Dentition at the age of 11. [file 6637180.f1.zip › 6637180.f1/Case 3 Dentition aged 11.png]
